# Impact on HIV-1 RNA Levels and Antibody Responses Following SARS-CoV-2 Vaccination in HIV-Infected Individuals

**DOI:** 10.3389/fimmu.2021.820126

**Published:** 2022-02-10

**Authors:** Vera Portillo, Chiara Fedeli, Pilar Ustero Alonso, Ianis Petignat, Ellen Cristina Mereles Costa, Adi Sulstarova, Cyril Jaksic, Sabine Yerly, Alexandra Calmy

**Affiliations:** ^1^ Division of Infectious Diseases, Geneva University Hospitals, Geneva, Switzerland; ^2^ Clinical Research Centre (CRC) & Division of Clinical Epidemiology, Department of Health and Community Medicine, University of Geneva and Geneva University Hospitals, Geneva, Switzerland; ^3^ Division of Laboratory Medicine, Geneva University Hospitals, and Centre for Emerging Viral Diseases and Laboratory of Virology, Geneva University Hospitals, Geneva, Switzerland; ^4^ Department of Medicine, Faculty of Medicine, University of Geneva, Geneva, Switzerland

**Keywords:** SARS-CoV-2, HIV, vaccination, PLWH, serology

## Abstract

This study aims to assess the immunological response and impact on virological control of the mRNA vaccines for severe acute respiratory syndrome coronavirus 2 (SARS-CoV-2) among people living with HIV (PLWH). In this single-center observational study, all PLWH were offered vaccination with mRNA1273 or BNT162b2. Both anti-N and anti-S1-receptor binding domain (RBD) antibodies were measured together with HIV-1 RNA levels after the first dose (M0) and then at 1 (M1), 2 (M2) and 6 (M6) months later. A total of 131 individuals (median age: 54 years [IQR: 47.0-60.5]; male: 70.2%; median baseline CD4 T-cell: 602/µl [IQR 445.0-825.5]; median nadir CD4 T-cells 223/µl [IQR 111.0-330.0]) were included. All participants were positive for anti-RBD antibodies at 30 days, 60 days and 6 months after the first dose, with no statistical difference between those with HIV-1 RNA below or >20 copies/ml. HIV-1 RNA data were collected for 128 patients at baseline and 30 days after the first dose; for 124 individuals, 30 days after the second dose; and for 83 patients, 6 months after the first dose. Nineteen (14.8%) of 128 had detectable HIV-1 RNA (>20 copies/ml) at M0, 13/128 (10.2%) at M1 (among which 5 were newly detectable), 15/124 (12.1%) at M2 (among which 5 were newly detectable), and 8/83 (9.6%) at M6. No serious adverse effects were reported. All participants elicited antibodies after two doses of mRNA vaccines, with only a minor impact on HIV-1 RNA levels over a 6-month period.

## Introduction

As of November 2021, more than 250 million people have been infected by the severe acute respiratory syndrome coronavirus-2 (SARS-CoV-2) (1). Information regarding COVID-19 prognosis in the 38 million people living with HIV (PLWH) suggests that they are at higher risk of a more severe clinical course of the disease (2–6). A strong protection is therefore warranted in this specific population. During early 2021, the vaccines mRNA1273 (Moderna/Spikevax) and BNT162b2 (BioNTech/Pfizer) were approved in Switzerland. Preliminary results of serological studies on PLWH showed a good serological response after the second dose for most individuals (7, 8), although it was unknown if this response would be sustained in the mid-term. Past experience with two doses of AS03-adjuvanted influenza vaccine resulted in a strong serological response, but also in a transient effect on HIV-1 RNA levels in participants with a previously well-controlled infection (9).

We aimed to assess the humoral response to the SARS-CoV-2 vaccine over 6 months in PLWH after two doses of vaccine. Secondary objectives were to measure the impact of the SARS-CoV-2 vaccine on HIV-1 RNA levels at one and two months, followed by data recorded in the patient’s file thereafter, and vaccine safety.

## Methods

We conducted an observational, open-label study nested in the Swiss HIV Cohort Study (SHCS) at the HIV/AIDS Unit of our institution starting on 26 January 2021. Participants received two doses of SARS-CoV-2 mRNA vaccine 4 weeks apart, as recommended by the Swiss Federal Office of Public Health (FOPH) (10), either mRNA1273 or BNT162b2, according to availability. Inclusion criteria were PLWH aged 18 years or over and enrolled in the SHCS. A group of healthy volunteers was recruited during the same period in our hospital and all received mRNA1273 vaccine. Recruitment was done using a pragmatic approach: every individual fulfilling vaccination criteria and consenting to routine vaccination according to priorities established by the FOPH were eligible and asked to sign a written consent form.

We collected blood samples at the time of the first (M0) and second (M1) dose of the vaccine, 30 days after the second dose (M2), and 6 months after the first dose (M6) for both PLWH and the healthy volunteers. We measured anti-SARS-CoV-2 S1-receptor-binding domain (RBD) antibodies (anti-RBD antibodies) and anti-SARS-CoV-2 N total antibodies (anti-N antibodies), using Roche Elecsys^®^ (Roche Diagnostics, Switzerland) according to the manufacturer’s instructions (11, 12). Results for anti-RBD were normalized using WHO standard (NIBSC, 20/136) and reported in IU/ml. Cut-off for positivity was set-up at > 1.1 IU/ml for anti-RBD and at index > 1.1 for anti-N. For PLWH, age, CDC stage, and nadir CD4 T-cell count were extracted from the SHCS database. Baseline CD4 T-cell counts were extracted from the hospital electronic medical records. We systematically measured HIV-1 RNA at M0, M1 and M2. Thereafter, we collected HIV-1 RNA levels (M6) as per standard of care. We used the COBAS 6800 (Roche Diagnostics) to assess HIV-1 RNA levels. A detectable viral load was defined as >20 copies/ml (13). Vaccination side effects were self-reported one month after each dose. Participants were followed until 6 months to identify any potential post-vaccination infections or late side effects. Data were collected using a paper-based case report form and stored in a password-secure Excel sheet.

### Statistical Analysis

Categorical variables are presented as counts and relative proportions. Continuous variables are expressed as means with standard deviations (SD) and/or as the median and interquartile range (IQR). Group comparisons of continuous variables were conducted using Welch’s t-tests. No missing data imputation was conducted. Statistical analyses were performed using R software, version 4.1.0. The protocol was approved by the Geneva cantonal ethics committee on 9 March 2021 (no. 2021-00491).

## Results

**Figure 1:**
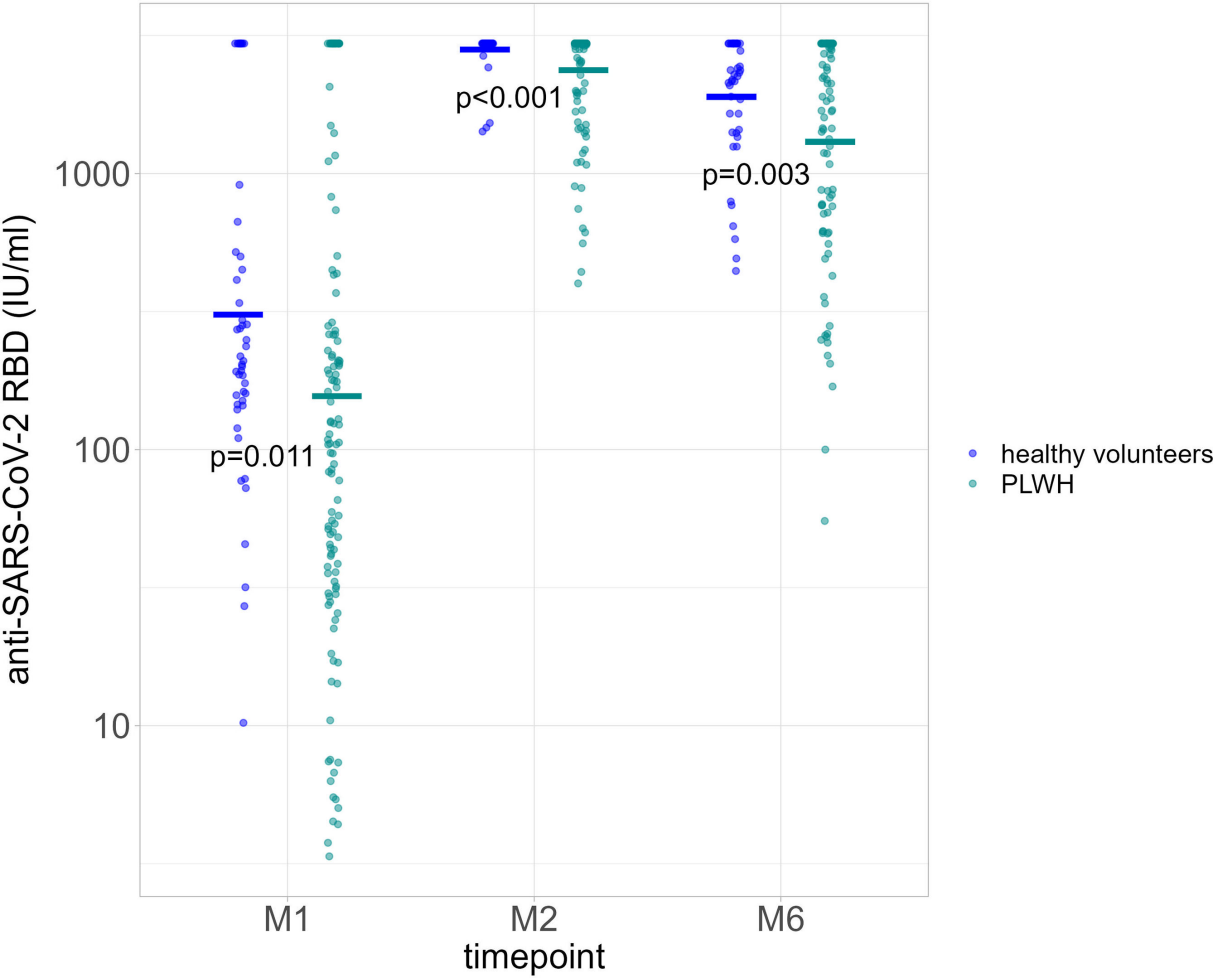
Quantification of anti-RBD Ig (GMT) in healthy volunteers (blue dots) and people living with HIV (PLWH, green dots) at each time point. P value significant if <0.05. RBD, receptor binding domain; GMT, geometric mean titers; PLWH, people living with HIV; M1, 30 days after the first vaccine dose; M2, 60 days after the first vaccine dose; M6, 6 months after the first vaccine dose.

A total of 131 PLWH (median age 54 years [IQR 47.0-60.5]; 70.2% male; median nadir CD4 T-cells 223 cells/µl [IQR: 111.0-330.0]) were included in the analysis (**Table 1**). mRNA-1273 was administered to 78 participants (59.5%) and BNT162b2 to 53 participants (40.5%). The group of healthy volunteers was composed of 49 participants (median age 30 years [IQR 27.0–34.0]), of which 30/49 (61.2%) were male. For PLWH, anti-RBD serology was available for 129 participants at M1, 124 at M2, and 95 at M6, and for 49 healthy volunteers at M1, 48 at M2, and 44 at M6. Geometric mean titers (GMT) for PLWH were 156.1 IU/ml (95% CI110.8-220.0), 2372.0 IU/ml (95% CI 2192.3-2566.4), and 1303.4 IU/ml (95% CI 1075.7-1579.2)) at M1, M2 and M6, respectively. For the healthy volunteers, GMT were 308.5 IU/ml (95% CI 206.6-460-6) at M1, 2815.6 IU/ml (95% CI 2677.9-2960.3) at M2, and 1896.5 IU/ml (95% CI 1611.4-2232.1) at M6. GMT values were statistically higher for the healthy volunteers at each time point (**Figure 1**).

**Table 1:** Baseline characteristics for PLWH.

	N	PLWH
Total population	131	131
**Age, years**, median [IQR]	131	54 [47.0-60.5]
**Gender**, n (%)	131	
Male		92 (70.2)
Female		39 (29.8)
**HIV stage Category**, n (%)	128	
A		80 (62.5)
B		25 (19.5)
C		23 (18.0)
**Nadir CD4**, median [IQR]		
Total count (cells/µl)	129	223 [111.0-330.0]
Percentage	128	21 [11.8-31.0]
**Baseline CD4**, median [IQR]	131	
Total count (cells/µl)		602 [455.0-825.5]
Percentage		35 [27.5-42.0]
**HIV-1 RNA**, n (%)	128	
Detectable		19 (14.8)
Undetectable (≤ 20 copies/ml)		109 (85.2)
**Anti-N SARS-CoV-2**, n (%)	130	
Positive		28 (21.5)
Negative		102 (78.5)

PLWH, people living with HIV; IQR, interquartile range.

**Figure 2:**
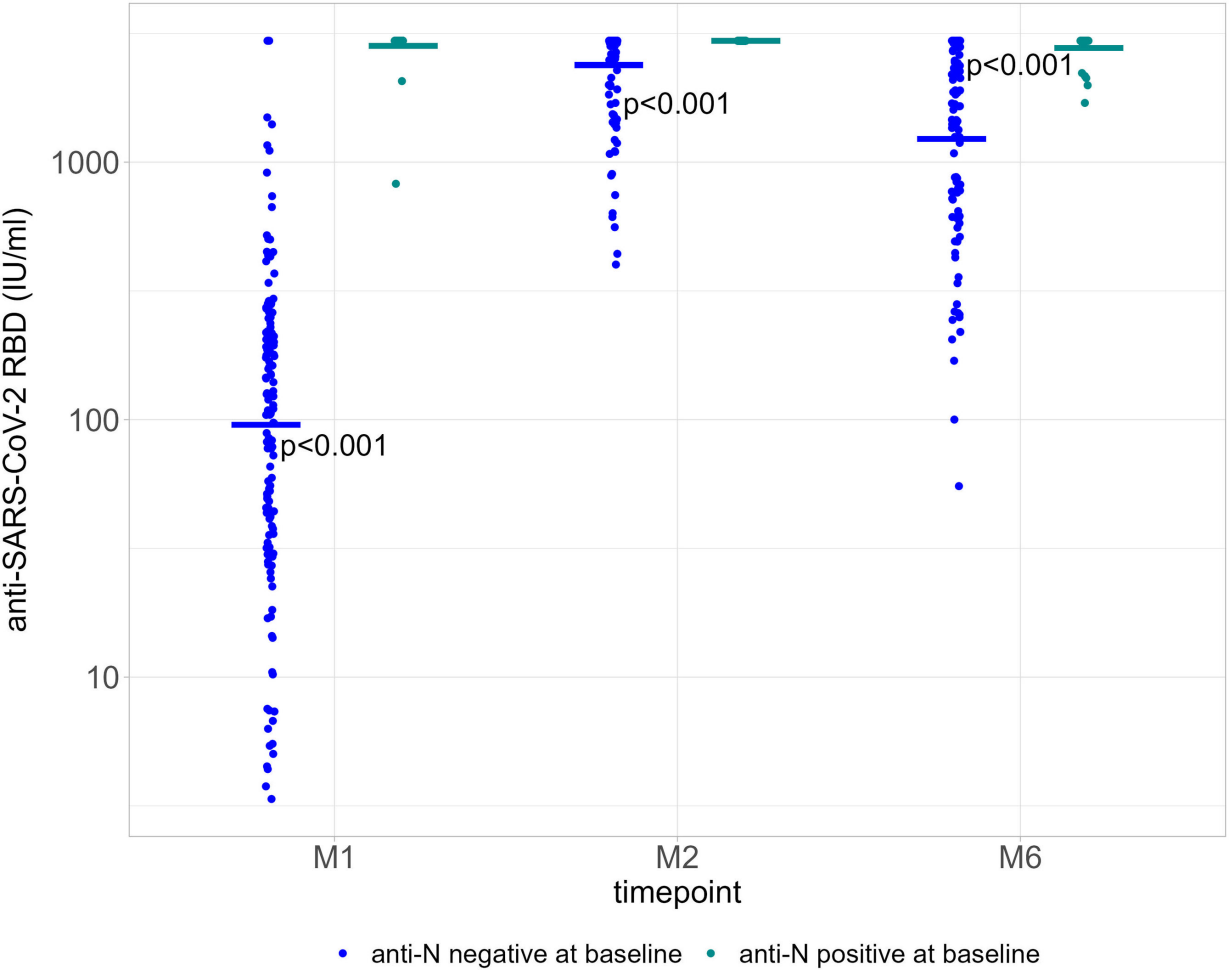
Quantification of anti-RBD Ig (GMT) according to baseline anti-N positivity (negative, blue dots; positive, green dots) at each time point. P value significant if <0.05. RBD, receptor binding domain; GMT, geometric mean titers; M1, 30 days after the first vaccine dose; M2, 60 days after the first vaccine dose; M6, 6 months after the first vaccine dose.

**Figure 3:**
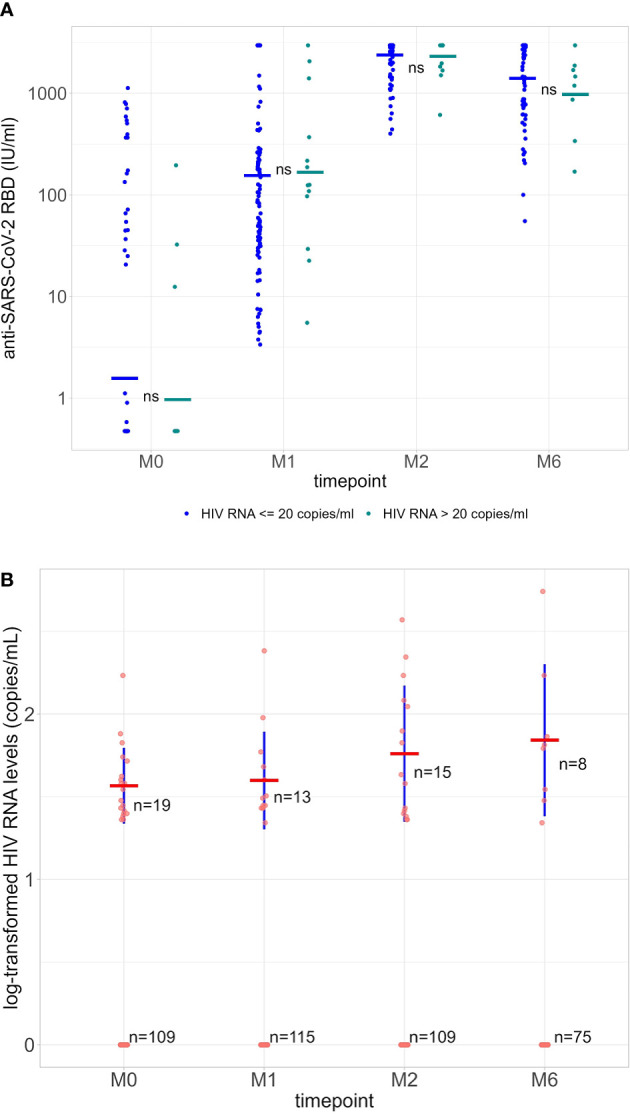
**(A)** Quantification of anti-RBD Ig (GMT) in HIV-1 undetectable patients (≤20 copies/ml, blue dots) and HIV-1 detectable patients (>20 copies/ml, green dots) at each time point. ns = not significant (P-value >0.05). **(B)** Log-transformed values of HIV-1 RNA levels at each time point. Average values (red) and standard deviations (blue) were calculated only using detectable HIV-1 RNA levels (>20 copies/ml). Differences are not statistically significant (P>0.05). RBD, receptor binding domain; GMT, geometric mean titers; M0, at the first dose; M1, 30 days after the first vaccine dose; M2, 60 days after the first vaccine dose; M6, 6 months after the first vaccine dose.

We compared GMT for PLWH based on anti-N positivity (indicative of a previous SARS-CoV-2 infection) at baseline and at each time point. A history of previous COVID-19 disease was not reported. GMT were consistently higher among those with a positive anti-N result at baseline, 2791.67 IU/ml [95% CI 2535.1-3074.2] for 28/28 participants at M1; 2960 IU/ml [no variance] for 27/28 participants at M2; and 2759.0 IU/ml [95% CI 2576.7-2954.2] for 23/28 participants at M6). Among those with a negative anti-N at baseline, GMT were 70.7 IU/ml (95% CI 53.5-93.5) for 100/102 participants at M1, 2233.2 IU/ml (95% CI 2023.7-2464.4) for 96/102 participants at M2, and 1023.6 IU/ml (95% CI 813.6-1287.9) for 71/102 participants at M6 (**Figure 2**). Serological responses were not significantly different between participants with more or less than 20 HIV-1 RNA copies/ml at M1, M2 and M6 (**Figure 3A**).

HIV-1 RNA was >20 copies/ml at baseline in 19/128 participants (14.8%; median, 34 copies/ml [IQR 24.5-46.0]; all <200 copies/ml), in 13/128 (10.2%; median, 30 copies/ml [IQR 26.0-47.0]; one >200 copies/ml) at M1, in 15/124 (12.1%; median, 42 copies/ml [IQR 24.5-115.0]; two >200 copies/ml) at M2, and in 8/83 (9.6%; median, 62.5 copies/ml [IQR 32.8-96.5]; one >200 copies/ml) at M6. Log-transformed values of HIV-1 RNA levels at each M0, M1, M2 and M6 are shown in **Figure 3B**.

**Figure 4:**
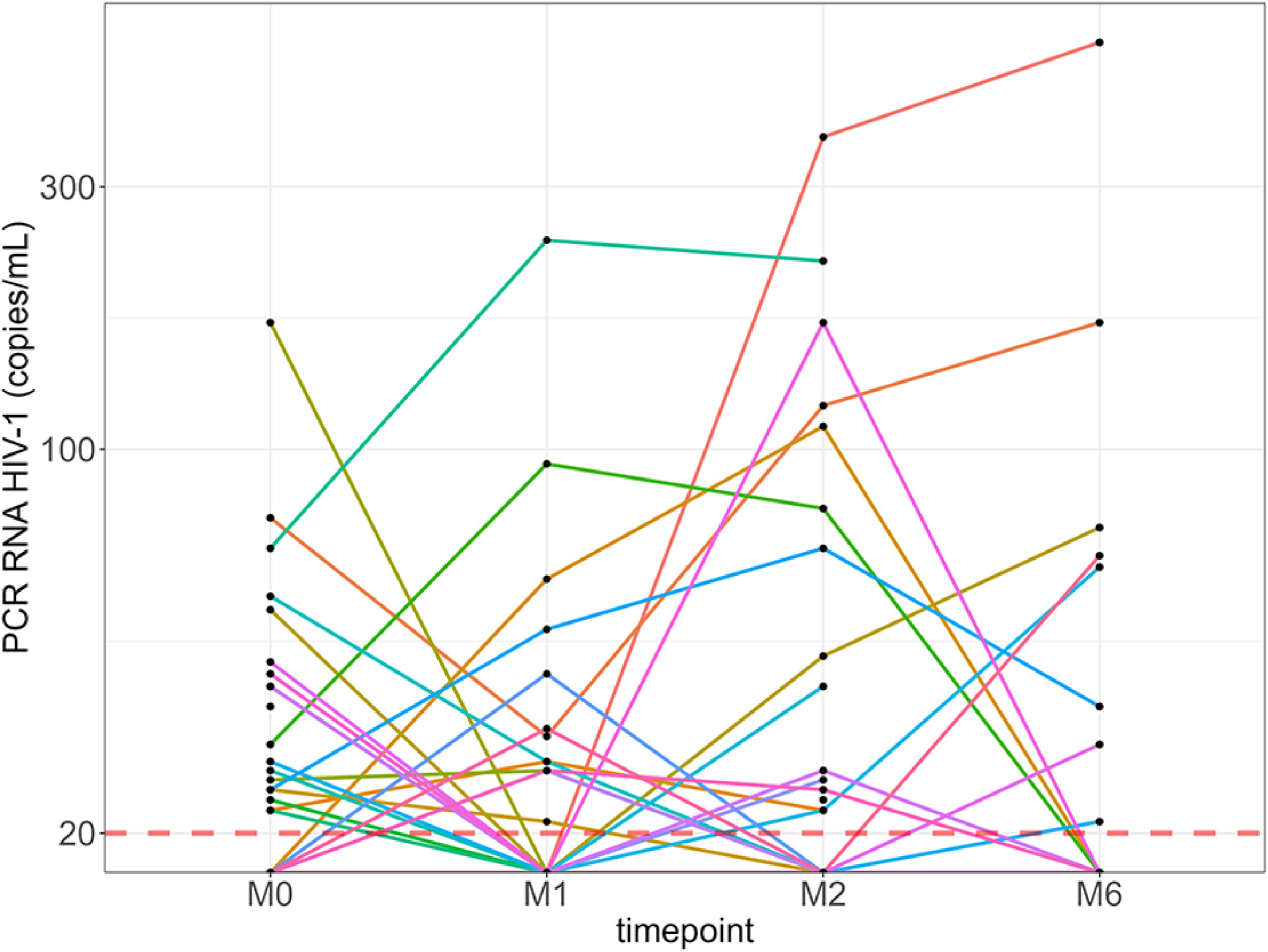
Kinetics of HIV-1 RNA levels of each individual with detectable levels over 6 months. Red line: HIV-1 RNA quantification threshold (20 copies/ml). M0, at the first dose; M1, 30 days after the first vaccine dose; M2, 60 days after the first vaccine dose; M6, 6 months after the first vaccine dose.

Among the 109/128 participants with HIV-1 RNA levels ≤20 copies/ml at M0, 5/109 (4.6%) became newly detectable at M1 (all ≤200 copies/ml), of which two remained detectable (24 and 110 copies/ml) at M2, becoming undetectable thereafter. Of the 115/128 participants with ≤20 copies/ml at M1, 7/110 (6.4%) had detectable values by M2 (range, 22-370 copies/ml), of which 3/5 had HIV-1 RNA levels >20 copies/ml (only one >200 copies/ml) at M6 (**Figure 4**). Overall, the percentage of participants with HIV-1 RNA levels >20 copies/ml did not change significantly at each time point.

Two participants in the PLWH group developed COVID-19 post-vaccination (2 days after the first dose and 3 months after the second dose), both with mild disease. Regarding side effects in the PLWH group, 62.0% reported mild side effects, 16.8% moderate, and one reported severe fatigue after the first dose. After the second dose, 54.0% reported mild side effects, 24.6% moderate, and one patient reported severe pain at the injection site. No serious adverse effects were reported.

## Discussion

PLWH on antiretroviral drugs elicited satisfactory anti-RBD antibodies titers up to 4 months after two doses of SARS-CoV-2 mRNA vaccine with no safety concerns, similar to data reported in other cohorts for this population (7, 8). The humoral response was significantly lower at any time point when compared to an unmatched group of healthy volunteers, despite an overall high CD4 T-cell count and good virological suppression. While we did not measure neutralizing antibodies (NAs), other studies have shown a high correlation between RBD-IgG antibodies and NAs (14). The humoral response elicited by these vaccines has been shown to be insufficient to protect severely immunosuppressed populations who may need additional doses, including PLWH with low CD4 T-cell count (15–18). A vaccine booster is being increasingly recommended in several countries for high-risk populations (19). In this respect, PLWH are considered to be such a population by the World Health Organization, with a potentially 30% higher risk of hospitalization (20). In our cohort, only one patient had a breakthrough SARS-CoV-2 infection.

The safety of mRNA vaccines on HIV-1 RNA levels warrants attention as HIV viral suppression is a marker of antiretroviral therapy success. While we observed detectable HIV-RNA values during the 6-month study period, only one participant presented an HIV-RNA value >200 copies 6 months after the first vaccine administration. No major serious adverse events were observed. Participants included in this cohort will continue to be monitored.

Our study has some limitations. First, our participants had high CD4 T-cell counts and good virological control as they were all under an effective antiretroviral treatment. Therefore, our results are not generalizable to all PLWH. Second, all participants from the control group received the mRNA-1273 vaccine, which has been shown to have higher immunogenicity in one study (21).

In conclusion, among PLWH routinely and regularly followed up in a specialized consultation in Switzerland, participants elicited a good anti-RBD antibodies response after the first two doses of mRNA vaccines, with only a minor impact on RNA-HIV-1 levels at most.

## Data Availability Statement

The original contributions presented in the study are included in the article/supplementary material. Further inquiries can be directed to the corresponding author.

## Ethics Statement

The studies involving human participants were reviewed and approved by Commission Cantonale d’Ethique de la Recherche sur l’être humain (CCER). The patients/participants provided their written informed consent to participate in this study.

## Author Contributions

VP, PU, CF, AC, and CJ had full access to all of the data in the study and take responsibility for the integrity of the data and the accuracy of the data analysis. Concept and design: VP and AC. Drafting of the manuscript: VP, PU, CF, and IP. Critical revision of the manuscript for important intellectual content: All authors. Statistical analysis: CJ. Obtained funding: VP and AC. Administrative, technical, or material support: SY and CF. Overall study responsibility: AC. All authors contributed to the article and approved the submitted version.

## Funding

The COVAC-HIV study was funded by the HIV/AIDS Unit (Division of Infectious Diseases, Geneva University Hospitals) and the Projet de recherche et développement (PRD) of Geneva University Hospitals (grant ID # 2021-00491).

## Conflict of Interest

The authors declare that the research was conducted in the absence of any commercial or financial relationships that could be construed as a potential conflict of interest.

## Publisher’s Note

All claims expressed in this article are solely those of the authors and do not necessarily represent those of their affiliated organizations, or those of the publisher, the editors and the reviewers. Any product that may be evaluated in this article, or claim that may be made by its manufacturer, is not guaranteed or endorsed by the publisher.
